# Biodegradation Studies of Novel Fluorinated Di-Vinyl Urethane Monomers and Interaction of Biological Elements with Their Polymerized Films

**DOI:** 10.3390/polym9080365

**Published:** 2017-08-17

**Authors:** Yasaman Delaviz, Meilin Yang, J. Paul Santerre

**Affiliations:** 1 Institute of Biomaterials and Biomedical Engineering, University of Toronto, Toronto, ON M5G 1M1, Canada; y.delaviz@mail.utoronto.ca; 2 Faculty of Dentistry, University of Toronto, Toronto, ON M5G 1G6, Canada; Meilin.Yang@dentistry.utoronto.ca

**Keywords:** fluorinated monomers, urethane, methacrylates, albumin, degradation, resin composites, proteins, surfaces

## Abstract

The monomeric components of resin composites in dental restorative materials are susceptible to hydrolysis in the oral cavity. The main objective of this study was to assess the bio-stability of fluorinated urethane dimethacrylates and determine the nature of fluoro-chemistry interactions with protein and bacterial adhesion (both sources of hydrolytic activity) onto cured resin. Degradation studies were performed in the presence of either albumin (in a mildly alkaline pH) or cholesterol esterase (CE). The surface chemistry of the polymers was assessed by water contact angle measurements, pre- and post- incubation with albumin. Adhesion of *Streptococcus mutans* to cured resin was investigated. The fluorinated monomers were more stable against degradation when compared to the commercial monomer bisphenol A-diglycidyl methacrylate (BisGMA). While fluorinated monomers showed hydrolytic stability with respect to CE, all fluorinated monomers underwent some degree of degradation with albumin. The fluoro-chemistry did not reduce protein and/or bacterial adhesion onto the surface, however post incubation with albumin, the fluorinated surfaces still presented hydrophobic character as determined by the high contact angle values ranging from 79° to 86°. These monomers could potentially be used to increase the hydrophobicity of polymeric composites and provide a means to moderate esterolytic degradation associated with the monomeric component of the polymers within the oral cavity.

## 1. Introduction

Resin composites are among the most extensively used dental restorative materials despite having limited longevity in the oral cavity [1]. Resin composites are susceptible to hydrolysis, and the process is further catalyzed by salivary and bacterial derived enzymes [2,3,4]. Human saliva exhibits CE-like and pseudocholinesterase (PCE)-like hydrolase activities [2]. The esterase activity in saliva originates from different sources which includes epithelial cells, salivary glands, inflammatory response, and bacteria [5]. The highest fraction of esterase activity isolated from human saliva was found to be a mixture of proteins, among which albumin was identified as the leading protein with esterase activity that degrades BisGMA [4]. The esterase activity of albumin was enhanced with the formation of a complex containing Zinc-α2-glycoprotein, which can be simulated with slightly alkaline conditions [4]. *Streptococcus mutans* is another relevant source of esterase activity in the oral cavity that degrades resin composites and adhesive systems [3]. The rationale for improving the bio-stability of resin composites is thus supported by their degradation in the oral environment. In this study, both CE and albumin (in mildly alkaline environment) are used as physiologically relevant enzyme preparations to test the bio-stability of the newly synthesized fluorinated urethane monomers, and compare them to commercial BisGMA monomer.

The chemical composition of the monomers can ultimately define the bio-stability of the polymeric composites that arise from their polymerization. The enhanced stability observed with urethane-modified BisGMA when compared to BisGMA is believed to be facilitated by hydrogen bonded bridges between the urethane groups and the hydrolysable ester group [6]. As a result, a number of different urethane modified methacrylates have been synthesized [7,8,9,10]. Polar groups such as urethanes, esters, amides, and ethers are hydrophilic in nature and result in greater water sorption. To reduce water adsorption, pendant hydrophobic substituents can be incorporated into the backbone [11]. Another approach involves the use of fluorine-substitutes in the backbone or as side chains [9,12,13,14]. In addition to its hydrophobicity, fluorocarbon chains are bulkier than hydrocarbons [15], which can introduce steric hindrance. A number of fluoropolymers have presented greater resistance against bacterial adhesion due to their low surface energy [16,17,18,19]. However, such resistance to adhesion is not observed with all fluoropolymers, as *Streptococcus gordonii* and *Streptococcus mutans* have been shown to adhere to polytetrafluoroethylene (PTFE) [20,21].

**Figure 1:**
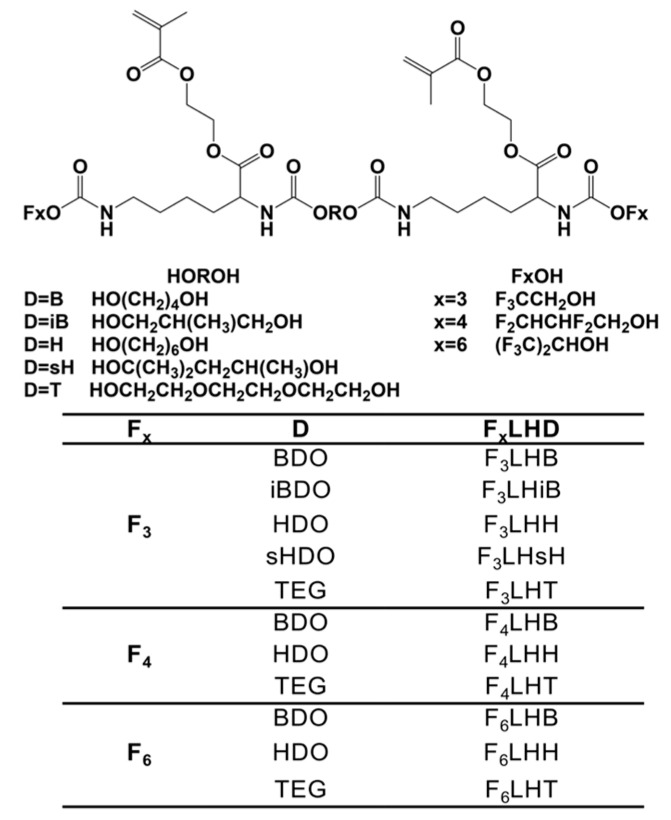
Schematic and nomenclatures of fluorinated monomers (F_x_LHD).

New fluorinated urethane dimethacrylate monomers were synthesized for use in dental resin composites. The chemical structure of the monomers is provided in Figure 1. The monomers were synthesized using short chain ether and non-ether diols, perfluoroalcohols and lysine diisocyanate (LDI). The fluorinated groups have the potential to repel water and shield hydrolytically sensitive esters, while the urethanes generated from the isocyanates have the potential to yield H–bonding with the esters, further delaying the hydrolysis of ester moieties. Methacrylate moieties were incorporated using 2-hydroxyethyl methacrylate (HEMA) to allow for in situ vinyl polymerization chemistry, which is needed for practical use. The main objective of the present work was to investigate the protein based hydrolytic processes that could interact with different dimethacrylate urethane monomers containing hydrophobic fluorine groups in the vicinity of the hydrolytically sensitive chemical linkages, as illustrated in Figure 1. Since the susceptible linkages to hydrolysis in the polymers is equivalent to that of the monomeric form (e.g., the esters) in its formulations, degradation analysis is performed with the monomers in solution in order to achieve an accelerated investigation of differences between the susceptibility of monomeric components to degradation [4]. Degradation analysis using the monomers also reduces the numbers of parameters that must be controlled for which influence the degradation profile, this includes surface area, degree of filler loading, degree of polymerization, and extent of crosslinking [4]. Furthermore, it was a goal to characterize the influence of the fluorinated chemistry on bacterial adhesion behavior, when the bacteria were interacting with polymer resins generated from the fluorinated-urethane monomers.

## 2. Materials and Methods

All materials were purchased from Sigma-Aldrich Inc. (St. Louis, MO, USA) unless otherwise stated. 

### 2.1. Synthesis of Fluorinated Monomers

Fluorinated hydrophobic monomers (F_x_LHD) (see structure and nomenclature in Figure 1) were synthesized as previously described [22] using short chain ether and non-ether diols, perfluoroalcohols, and LDI with HEMA substituted for the methyl ester of LDI units to generate a di-vinyl monomer. The following reagents were used for the synthesis: dichloromethane (anhydrous, purity 99.8%), 1,4-butanediol (purity ≥ 99%), 2-methyl-1,3-propanediol (purity ≥ 99%), 2-methyl-2,4-pentanediol (purity ≥ 99%), triethylene glycol (purity 99%), 1,6-hexanediol (purity 99%), HEMA (purity 97%), acetone (purity 99.5%), chloroform (ACS reagent), dibutyltin dilaurate (purity 95%), diethyl ether, 4-(dimethylamino) pyridine (purity ≥ 99%), *N*,*N*-dimethylacetamide anhydrous (purity 99.8%), 4-methoxyphenol (purity 99%), 2,2,2-trifluoroethanol (purity ≥ 99%), 2,2,3,3-tetrafluoro-1-propanol (purity 98%), 1,1,1,3,3,3-hexafluoro-2-propanol (purity ≥ 99%), hexane (purity ≥ 95%), trifluoroacetic acid (purity 99%), diethyl ether (purity 99.8%), tert-butanol (purity 99.5%), 1-ethyl-3-(3-dimethylamino-propyl) carbodiimide·HCl (Advanced ChemTech CreoSalus, Louisville, KY, USA), LDI (Daming Changda Co., Ltd., Daming, China), and alcalase (EMD Chemicals Inc., San Diego, CA, USA, activity 3.0264 U/mL, batch No. D00095203).

### 2.2. Hydrolysis of Monomers in the Presence of Human Serum Albumin (HSA)

Degradation studies were performed in protein solutions of 0.35 g·L^−1^ non-denatured HSA (Calbiochem 126654, purchased from EMD Chemicals Inc., San Diego, CA, USA) dissolved in a tris buffer (pH 8.8). The buffer contained 20 mM Trizma base, 2 mM calcium chloride (CaCl_2_), 5 mM magnesium chloride (MgCl_2_; BioShop, Burlington, ON, Canada), with pH adjusted to 8.8 using 0.1 N hydrochloric acid and 0.1 N sodium hydroxide (BioShop, Burlington, ON, Canada) [4]. The protein solution was filtered using a Millex-GP 0.22 μm syringe filters (Millipore, Bedford, MA, USA). Fluorinated monomers (F_x_LHD) (structure and nomenclature is described in Figure 1) and BisGMA (Esschem Inc., Linwood, PA, USA) were dissolved in HPLC grade methanol (MeOH; Caledon Laboratories Ltd., Georgetown, ON, Canada) separately at a concentration of 5 mM, and were then added to separate protein solutions to yield a final monomer concentration of 0.1 mM with 2 vol % MeOH. The solutions (*n* = 3) were then aliquoted into separate amber vials, with 0.5 mL of solution per vial for each time point, sealed and incubated at 37 °C. At specific time points (0, 1, 2, 3, 4, and 5 days), the corresponding vials were removed from the oven and 0.5 mL of MeOH was added to each vial and vortexed to denature the protein and cease the enzymatic activity. The solutions were then filtered using Amicon Ultra-0.5 centrifugal filtration units with 3 kDa molecular weight cut off (Millipore, Bedford, MA, USA) for 30 min at 14,000 rcs (MPW-65R Centrifuge, Med Instrument, Warsaw, Poland) and at 8 °C. The supernatant was collected and stored at −20 °C until analysis. The experiment was repeated three times with triplicates (*n* = 9).

### 2.3. Hydrolysis of Monomers in the Presence of CE

CE solution was prepared by dissolving the required amount of powder enzyme (CE, Toyobo Co. COE-313, Osaka, Japan) in Dulbecco’s phosphate-buffered saline (DPBS; Gibco, Grand Island, NY, USA). The CE activity was measured at 401 nm on a DU800 spectrophotometer (Beckman Coulter Inc., Fullerton, CA, USA), at pH 7.0 and 37 °C using 4-nitrophenylbutyrate (pNPB) as the substrate [23]. One unit of esterase activity was defined as generating 1 nmol/min of *p*-nitrophenol from pNPB at pH 7.0 [24]. Select synthesized fluorinated monomers (F_3_LHT, F_6_LHT) and a commercial control monomer BisGMA were dissolved in MeOH and diluted with DPBS to yield a final concentration of ~0.01 mM. From each monomer solution (~0.01 mM), 2 mL were added to 15 separate vials giving a total of 45 vials per monomer condition (*n* = 3). To each vial, CE solution was added to yield an activity of 5 units/mL, and the vials were incubated in an oven at 37 °C for 24 h. At specific time points (1, 4, 8, 10 and 24 h), 3 vials for each monomer condition were removed from the oven and 133 µL of MeOH was added to denature the enzyme and cease the esterolytic activity [5].

### 2.4. High Performance Liquid Chromatography (HPLC)

Degradation solutions were analyzed using a Waters^TM^ HPLC system (Mississauga, ON, Canada) equipped with a 600E multi-solvent delivery system and 996 photodiode array (PDA) detector connected to a data acquisition system, and signals were processed using Empower Software (Waters Corporation, Milford, CT, USA). Samples (50 μL) were injected into a Kinetex^TM^ C18 column (Phenomenex 00D-4462-E0, Torrance, CA, USA) using an 800 series Hamilton syringe (Hamilton Company, Reno, NV, USA). Solutions were eluted through the system at a flow rate of 1.0 mL/min with helium gas running through the mobile phase at a sparge rate of 60 mL/min. A gradient method was used beginning with 30:70 vol % of MeOH: 20 mM ammonium acetate (AnalaR, BDH Inc., Toronto, ON, Canada) buffer to 100% MeOH for 7 min. The peak areas for the F_x_LHD monomers were measured at a retention time of approximately 3 min and 215 nm h. For BisGMA, the gradient method was followed by elution with 100% MeOH for 8 min, and the peak area was measured for a retention time of 11 min at 280 nm. HPLC chromatograph for each monomer at day 0 is available in the supplementary information (Figures S1 and S2). Before every injection, the pressure was equilibrated by running the initial mobile phase (30 vol % MeOH-70 vol % buffer) for 5 min. When analyzing samples incubated in the presence of CE, a previously developed method consisting of Luna 5 µm C18 100A 250 × 4.60 mm column (Phenomenex, Torrance, CA, USA) was used [5].

### 2.5. Preparation of Polymerized Monomer Resins

Camphorquinone (CQ) and 2-(dimethylamino) ethyl methacrylate (DMAEM) were used as the photo-initiator system. For the polymerization of resins, 0.45 g of monomer was blended with 28.8 µL of initiator solution composed of 0.2 g CQ, 0.4 g DMAEM, and 3.6 mL DriSolv^®^ dichloromethane (EMD Millipore, Bedford, MA, USA). The solutions were left to mix overnight with aluminum foil wrapped around the vials to protect against pre-mature polymerization. The mixtures were formed in cylindrical Teflon^TM^ molds (3 mm diameter × 1 mm height or 5 mm diameter × 1 mm height) and the solvent was left to evaporate at room temperature for at least 15 min while being protected from direct light. Mylar^TM^ strips were placed over the Teflon^TM^ molds and polymerization was photo-initiated using a dental blue light, either a FLASHlite 2.0 or Sapphire plus (DenMat, Santa Maria, CA, USA), at room temperature for 1 min per side. The photo-polymerized cylindrical samples were subsequently post cured for 24 h in the oven at 60 ± 5 °C.

### 2.6. Water Contact Angle Measurement and Indirect Measurement of Protein Adhesion

Thin films of material were cured on glass slides (VWR International, Mississauga, ON, Canada) using the same initiator system and polymerization as described above. Slides were post cured in the oven at 60 ± 5 °C overnight, and allowed to reach room temperature prior to contact angle measurements. The advancing and receding contact angles of water on the film surfaces were determined using a contact angle goniometer (NRL Model 100-00, Ramé-Hart, Inc., Mountain Lakes, NJ, USA). Commercial monomers BisGMA and urethane dimethacrylate (UDMA; Esschem Inc., Linwood, PA, USA) were used as controls for relative comparison. Measurements were done by placing a droplet of MilliQ water using a micro-syringe on the polymers’ surfaces, and the contact angle was recorded for either side of the droplet. The average value was used for a single measurement. For each material, 3 polymer films were prepared with at least 5 drops per film recorded.

After contact angle measurements were recorded, the polymer films were exposed to 1 g·L^−1^ bovine serum albumin (BSA) solution prepared in DPBS for 45 min at room temperature. Samples were rinsed several times with distilled water to remove non-adhered proteins, and left in the fridge for 72 h. Polymer samples were removed from the fridge and allowed to reach room temperature prior to repeating the contact angle measurements. 

### 2.7. Direct Measurement of Protein Adhesion Assay

Cylindrical polymer specimens were first incubated in 70% ethanol overnight to extract potential unreacted monomer or initiator from the polymeric material. Subsequently, the ethanol was removed and samples were left to dry in the biosafety cabinet for 4 h. Samples were then incubated with 1 g·L^−1^ HSA in DPBS for 1 h at 37 °C, rinsed three times with DPBS and placed into new wells. The adsorbed proteins were then eluted at room temperature using 2% sodium dodecyl sulfate (SDS; BioShop, Burlington, ON, Canada) under gentle shaking on a shaker plate for 24 h. The resulting recovered solutions were assessed for total protein content using a micro bicinchoninic acid (BCA) protein assay (Thermo Scientific, Rockford, IL, USA).

### 2.8. Colony Forming Unit (CFU) Counts

Bacterial adhesion onto polymerized cylindrical specimens was assessed under static conditions. *Streptococcus mutans* UA159 stored in 20% glycerol broth at −80 °C were recovered and sub-cultured on Todd-Hewitt (TH) agar plates with 0.3% yeast extract (BioShop, Burlington, ON, Canada) and incubated for 48 h at 37 °C and 5% CO_2_. Todd-Hewitt yeast extract (THYE) broth was prepared from 30 g·L^−1^ TH and 3 g·L^−1^ yeast extract, with the addition of 18 g·L^−1^ agar (BioShop, Burlington, ON, Canada), when preparing THYE agar plates. Several well-isolated colonies were removed from the plate and inoculated into fresh THYE broth medium and incubated overnight at 37 °C and 5% CO_2_. The overnight solutions were diluted in a 1:10 dilution with THYE medium. After aging the specimens in DPBS for 48 h to condition the polymer surfaces and extract potential unreacted monomers from the samples, the cured cylindrical specimens were placed in a 96 well plate with 200 μL of the 1:10 diluted overnight solution. The specimens were incubated with bacteria for 24 h at 37 °C, upon which samples were rinsed three times with sterile DPBS to remove non-adhered bacteria cells. Subsequently, each specimen was added to a sterile container with DPBS and placed in an ultrasonic bath (Bransonic 2510 Ultrasonic cleaner, Danbury, CT, USA, 42 kHz ±6%) and sonicated for 1 min to recover the adhered bacteria [25,26]. The solutions were then serially diluted and plated onto THYE agar plates followed by 48 h of incubation at 37 °C and 5% CO_2_. The CFU of the disrupted biofilm cultures recovered were then counted after 48 h of incubation.

### 2.9. Statistical Analysis

All statistical analyses were performed using IBM SPSS program (IBM Corp., Armonk, NY, USA, version 20.0). One-way ANOVA was performed after verifying homogeneity of variance (Leven’s test) for albumin and *S. mutans* adhesion. For contact angle measurements, student’s *t*-test was used to determine the statistical significance of any difference between the mean values of pre- to post-incubation conditions for advancing and receding values. To compare the different polymeric surfaces, a Welch ANOVA was used with Games–Howell post-hoc analysis. For biodegradation studies, paired *t*-test was used to compare the percent residual of each monomer relative to its non-degraded concentration at different time points. One-way ANOVA with Tukey post-hoc analysis was performed after verifying homogeneity of variance (Leven’s test) to compare the percent residual for the different monomers. The significant threshold was set to ∝ = 0.05 for all analyses.

## 3. Results

**Figure 2:**
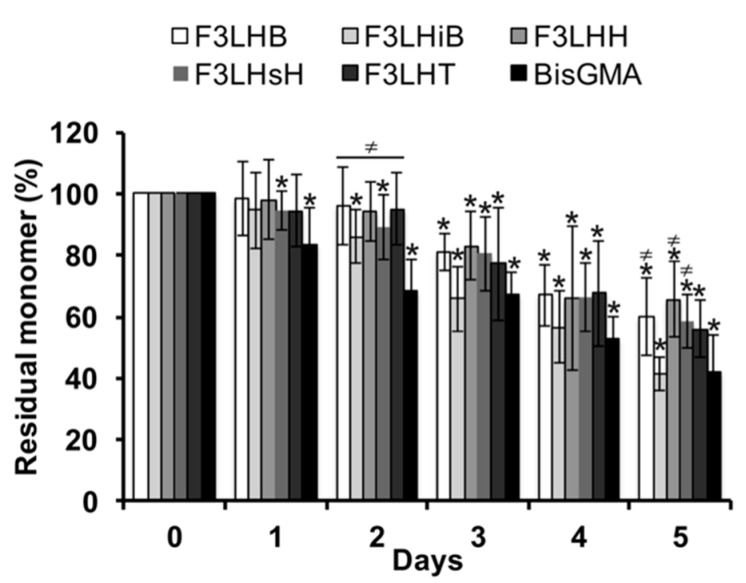
Monomer Degradation. Residual monomer in the biodegradation solution incubated with 0.35 g·L^−1^ HSA at pH 8.8 (*n* = 9, ±standard deviation (SD)). * Denotes significant difference (*p* < 0.05) from Day 0. For a given time point, ≠ denotes significant difference (*p* < 0.05) from BisGMA.

Over the course of the first two days of monomer degradation during incubation in HSA at pH 8.8, the level of native F_3_-type fluoro-monomer had remained close to 90%, whereas the BisGMA control had already dropped to less than 70% of the original monomer level, significantly (*p* < 0.05) lower than the F_3_-type fluoro-monomers (Figure 2). Significant loss of original monomer began at the 72 h time point for several of the F_3_ fluoro-monomers and showed a continued loss over time. After 5 days of incubation, the percent residual BisGMA was significantly lower (*p* < 0.05) than F_3_LHB, F_3_LHH, and F_3_LHsH, which had as much as 20% more residual monomer (Figure 2). 

**Figure 3:**
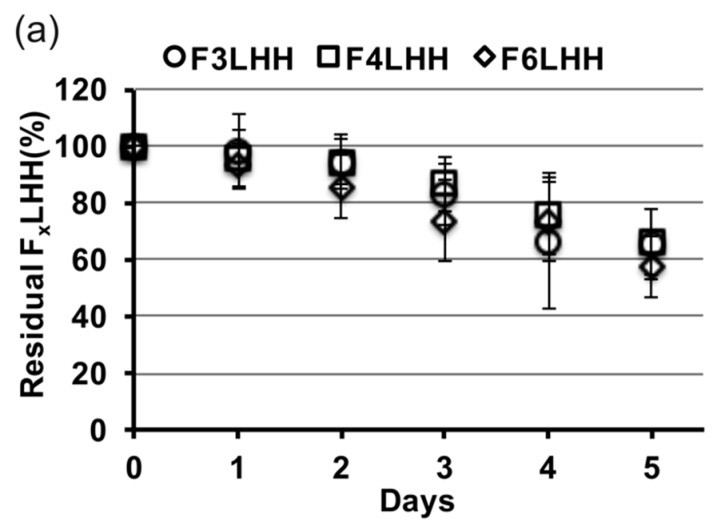

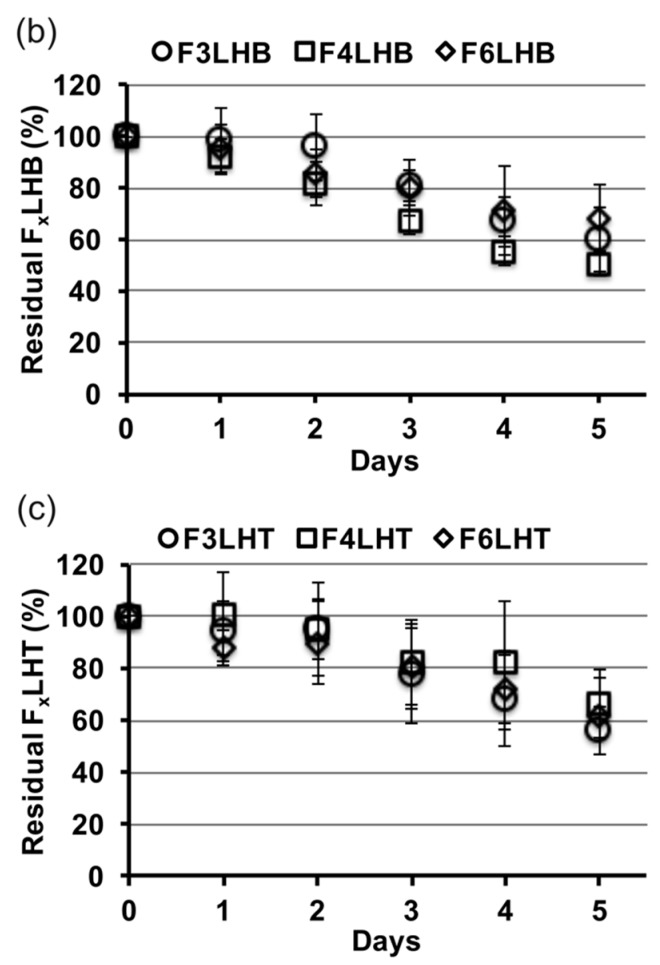
Residual F_x_LHD monomer in the biodegradation solution incubated with 0.35 g·L^−1^ HSA at pH 8.8 (*n* = 9, ±SD), arranged based on the nature of diol linkers: (**a**) hexane diol (F_x_LHH); (**b**) butane diol (F_x_LHB); and (**c**) triethylene glycol (F_x_LHT).

Figure 3 shows the relative slopes of all F_x_LHD monomers that underwent degradation in the presence of HSA at pH 8.8, from which degradation rates were calculated. The percent loss of monomer per day was greatest for BisGMA (12%, *R*^2^ = 0.957), followed by F_3_LHiB (11%, *R*^2^ = 0.958), F_4_LHB (10%, *R*^2^ = 0.985), F_3_LHT (8%, R^2^ = 0.905), F_6_LHH (8%, *R*^2^ = 0.963), F_3_LHsH (8%, *R*^2^ = 0.959), F_3_LHB (7%, *R*^2^ = 0.878), F_6_LHT (7%, *R*^2^ = 0.948), F_3_LHH (7%, *R*^2^ = 0.882), F_6_LHB (7%, *R*^2^ = 0.987), F_4_LHH (6%, *R*^2^ = 0.908), and F_4_LHT (5%, *R*^2^ = 0.837). Comparing the degradation profile of F_x_LHT to F_x_LHH monomers suggests that the hydrophobicity of the core does not significantly (*p* > 0.05) influence the degradation of the monomer components in solution (Figure 3). The F_x_LHT monomers contain the hydrophilic triethylene glycol and the F_x_LHH monomers contain the hydrophobic 1,6-hexane as the core (Figure 1). Increasing the fluorine-content from F_3_ to F_6_ was also not found to significantly (*p* > 0.05) alter the hydrolysis rate of the monomers in solution (Figure 3), although select combinations of fluoro and dihydroxyl spacers did yield up to two-times lower degradation rates than the commercial BisGMA.

**Figure 4:**
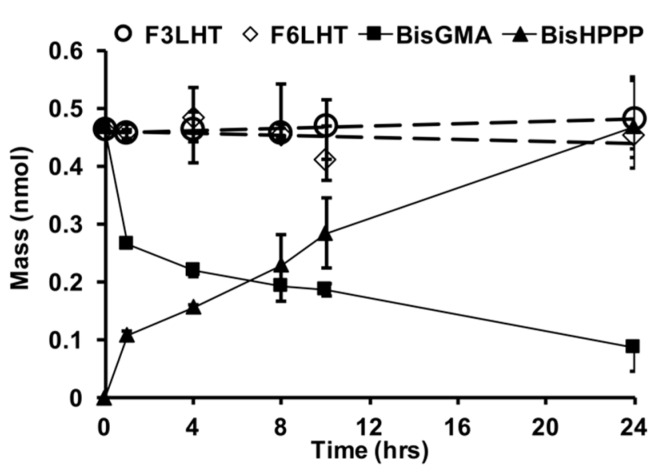
Hydrolytic degradation studies of two select fluorinated monomers (F_3_LHT and F_6_LHT) vs. BisGMA in the presence of 5 units/mL CE, at 37 °C (*n* = 3, ±SD).

Two of the more stable monomers were also examined for their relative stability towards CE (Figure 4), which is an enzyme-like activity that has been found in saliva to be highly active towards the commercial monomer BisGMA [2,4]. Within 24 h of exposure to CE, BisGMA had undergone degradation by 79% and produced equivalent molar amounts of bisphenol A bis (2,3-dihydroxypropyl) ether (BisHPPP), the terminal degradation by-product. However, F_3_LHT and F_6_LHT showed no visible biodegradation under the same in vitro condition (Figure 4). These findings further suggest that hydrogen bonding moieties and the hydrophobic fluorine atoms possibly were able to provide an initial protection of the ester linkage against degradation in DPBS with the presence of active CE.

The polymers generated from the monomers were then characterized for their interaction with water and protein. All of the fluorine containing polymers (pF_x_LHD) showed significantly higher (*p* < 0.05) advancing contact angles when compared to the two non-fluorinated polymer controls (Table 1). The change in advancing contact angle value for the F_x_LHD films upon exposure to albumin was less than ~5°, but more than 10° for the non-fluorinated controls. Similarly, the change in receding contact angles for the fluorinated materials were less than 5°, whereas the two controls showed significant drops in receding contact angles implying a substantially more hydrophilic substrate (16° for pBisGMA and 14° for pUDMA respectively). The hysteresis values before and after incubation with albumin were within a similar range of 30° to 40° for the pF_x_LHD films, suggesting that the heterogeneous nature of the polar and apolar group were not affected upon exposure to the protein solution. Contact angle hysteresis was greater on pF_x_LHD samples relative to the non-fluorinated controls pBisGMA and pUDMA, implying greater surface dynamics on the fluorinated samples. The elevated advancing values (79° to 86°) after incubation with albumin indicates that the hydrophobic fluorine groups are likely still present and influencing the surface after exposure to albumin, therefore maintaining the hydrophobic surface chemistry that was incorporated to enhance the stability against hydrolysis.

**Figure 5:**
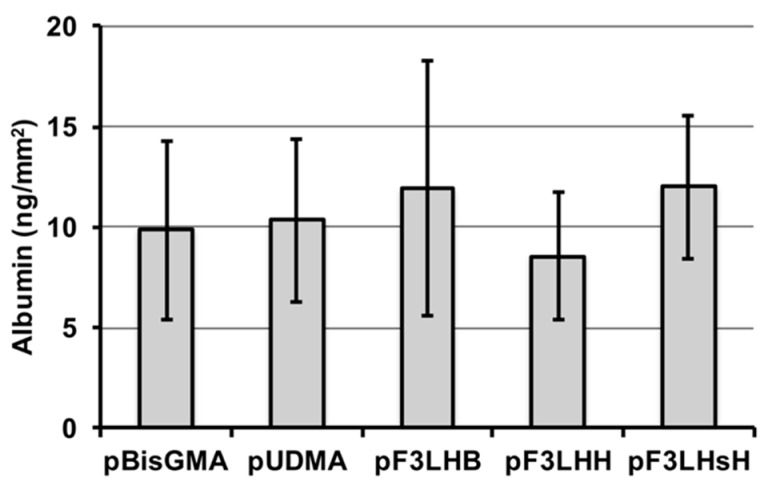
Albumin (HSA) adsorption following 1 h of incubation and 24 h of elution with 2% SDS solution for select fluorinated polymers and controls (*n* = 14, ±SD).

A select number of polymers made from F_x_LHD monomers containing hydrophobic cores (F_3_LHB, F_3_LHH, and F_3_LHsH) were used to determine whether the high advancing contact angle values observed on the pF_x_LHD films after exposure to albumin were related to surface chemistry or resistance to protein adhesion. HSA was found to adhere to all three fluorinated polymer surfaces, similar to the non-fluorinated polymer controls (Figure 5), suggesting that the high advancing values are related to hydrophobic chemical groups present at the surface rather than resistance to protein adsorption. 

**Figure 6:**
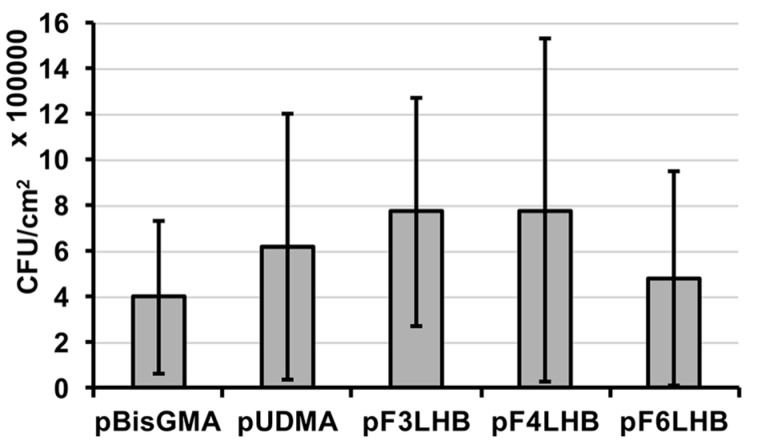
Adhered *S. mutans* after 24 h of incubation at 37 °C in 5% CO_2_ under static conditions for select fluorinated polymers and controls (*n* = 14, ±SD).

The influence of the polymers’ fluoro-content from the pendant structures (F_3_, F_4_, and F_6_) on bacterial adhesion was studied using polymers made from the F_x_LHB monomers and compared to the non-fluorinated controls. *S. mutans* were found to adhere to all studied polymer surfaces with no significant difference in the adhered CFU (Figure 6), suggesting that the fluoro-chemistry was inadequate in overcoming bacterial attachment to cured specimens.

## 4. Discussion

Fluorine atoms occupy a larger volume than hydrogen atoms because of the larger van der Waals radius. Fluoro-chains are also bulkier and more rigid than hydrocarbons [27], which may shield the susceptible ester linkages of the vinyl resin monomers from esterolytic degradation. In the mildly alkaline solution with the presence of HSA all fluoro-monomers underwent some degree of degradation, suggesting that the fluoro-chemistry was unable to fully shield all susceptible linkages from hydrolysis. However, the majority of the F_x_LHD monomers still presented greater stability against hydrolytic degradation when compared to the commercial monomer BisGMA. The biodegradation studies with albumin were done in a mildly alkaline environment (pH 8.8) as the esterolytic activity of albumin has been shown to become more potent at this pH [4]. The concentration of albumin used in the current study was selected to generate the equivalent of 5 units/mL CE-like activity with respect to pNPB to simulate the esterase activity found in human saliva [23].

In the presence of CE, the selected monomers F_3_LHT and F_6_LHT were found to have significantly greater stability against degradation when compared to BisGMA over 24 h. CE is an inflammatory cell derived enzyme that preferentially catalyzes the hydrolysis of long-chain fatty acid esters of cholesterol [2]. The catalytic reactivity of CE on a synthetic substrate depends on the ester side-chain, for example higher hydrolysis rates are observed with longer hydrocarbon side chains (butyrate vs. acetate) [2]. Components of human saliva exhibit CE-like behavior, which preferentially degrade the aromatic monomer BisGMA versus triethylene glycol dimethacrylate, another commercially used monomer, and similar behavior is also observed with pure CE [23]. Similar to BisGMA, triethylene glycol dimethacrylate has been shown to undergo complete hydrolysis within 25 h in the presence of human saliva derived esterase activity [5]. Despite the difference in chemical structure, both monomers contain relatively unhindered esters that readily undergo hydrolysis in the presence of esterases. UDMA, another commonly used monomer that contains urethane bonds, has greater stability against hydrolysis when compared to BisGMA. In human saliva, BisGMA undergoes complete hydrolysis by 48 h while after 72 h of incubation more than 70% of UDMA is still left in solution [28]. Work by Hagio et al. [28] has shown that modifying BisGMA by replacing the hydroxyl moieties with short hydrocarbon side chains coupled via a urethane bond increases the hydrolytic stability in the presence of human saliva [28]. The enhanced stability was attributed to the difference in molecular structure, such as steric hindrance and the addition of the urethane bonds. The faster hydrolysis observed with BisGMA in the current study may therefore be related to the unhindered ester bonds available for hydrolysis and substrate preference for CE, while greater stability with the F_x_LHD monomers may be resulting from the branched structure and bulky fluoro groups creating steric hindrance for the enzyme to act on the esters.

The resulting polymers are expected to have diverse surface chemistries within their individual surfaces as all of the polymerized monomers contain non-polar and polar moieties, the latter resulting from polar carbonyl and urethane bonds in the urethane based monomers and polar carbonyl and hydroxyl moieties in BisGMA. The higher hysteresis observed with the pF_x_LHD suggests a greater heterogeneity in the surface of the pF_x_LHD films, most likely related to having hydrophobic fluorine chemistry and polar moieties present within the monomers making up the surface. Changing the form of the fluoro-content from F_3_ to F_6_ structures did not further increase the advancing contact angle values. This may have possibly been due to a saturation of the effect for the fluorine chemistry relative to the presence of the remaining non-fluorinated chemistry resulting from the heterogeneity of other chemistries at the surfaces of the pF_x_LHD films. The high advancing contact angle values, even after exposure to albumin, suggest that the fluoro-chemistry’s influence remains present at the surface, which may provide a means to influence esterolytic activities generated by bacterial and salivary derived enzymes when interacting with resin composite.

All of the polymer surfaces studied were hydrophobic based on their advancing contact angle values being greater than 65° [29]. The adhesion of proteins and bacteria onto the polymer surfaces is highly affected by surface chemistry and wettability [30]. The adsorption process can involve different types of interaction including van der Waals, hydrogen bonding, hydrophobic and electrostatic interactions [31]. Hydrophobic interactions generally result in hydrophobic surfaces being more protein-adsorbent when compared to hydrophilic surfaces that are protected through repulsive solvation forces from the strongly bound water to the surface [32]. Similar to protein adhesion, hydrophobic surfaces are less resistant to bacterial adhesion [21]. However, super hydrophobic surfaces containing siloxane and fluorosiloxane have been shown to reduce protein adsorption and bacterial adhesion [33]. However, PTFE surfaces and resin composites with PTFE particle additives have been unable to inhibit protein adsorption and bacterial adhesion [20,21]. Similar to the PTFE surfaces, albumin was found to adhere to the pF_x_LHD surfaces. Proteins adsorb to polymer surfaces in different conformations and orientations depending on the surface chemistry and physical properties [31]. The high advancing contact angle values after exposure to BSA for the fluorinated surfaces suggests that the manner in which the proteins have adsorbed to the pF_x_LHD surfaces is different from that of the non-fluorinated controls. One hypothesis is that along with the hydrophobic fluoro chemistry at the polymer surface, BSA may be selectively interacting with the polar groups on the F_x_LHD derived polymers and enabling a hydrophobic presentation of the protein to the water droplet. In contrast, the hydrophobic interactions between the polymeric material and protein may be more dominant on the non-fluorinated surfaces with the protein’s polar groups being presented towards the water droplet, resulting in a more hydrophilic surface with significantly lower advancing and receding contact angle values post incubation. With such a differentiated interaction of albumin at the fluorinated polymer surfaces in play, then it becomes possible to rationalize the divergent outcomes of the esterolytic activities with the fluorinated monomer components observed in the earlier studies, and specifically those of albumin, as conformational structure is critically important to its hydrolytic activity in mildly basic conditions [4]. More precisely, the albumin associated esterase activity is dependent on the ligand binding sites of Tyr-411 and Arg-410 [34]. A substrate combines rapidly and reversibly to Tyr-411 and Arg-410, and the hydroxyl group of Ser-489 and the aromatic residue of Phe-488 help to stabilize the albumin-substrate intermediate. Both the arginine and serine amino-acid moieties are polar and could be hindered in their interaction with polar urethane groups of the fluorinated monomers. In combination with the water repelling character of the surfaces generated from the polymerized fluorinated monomers, even in the presence of albumin (Table 1), hydrolytic reactions would be hindered for the monomeric components as seen in Figure 2 and Figure 3 for albumin associated esterase activity.

Salivary proteins and microorganisms attach to restorative materials in the oral cavity [35,36,37,38]. Growth of *S. mutans* on resin composites has been known to roughen the surface of these restorative materials [39], and more recently *S. mutans* were shown to have esterolytic activity at levels that degrade resin composites [3]. Therefore, any means that may reduce esterolytic attack and alter the nature of protein presentation at the surface may potentially improve the longevity of the restorative material.

In the current study, the fluoro-chains incorporated were unable to reduce bacterial adhesion in vitro, with no significant (*p* > 0.05) difference in CFU count observed between the fluorinated and non-fluorinated surfaces. Adhered bacteria were removed from the surface by sonication using an ultrasonic bath, a technique commonly used for dislodging bacterial aggregates through physical, chemical and mechanical effects of acoustic cavitation, with the use of moderate frequencies between ~20 and 40 kHz [40]. Although these polymer surfaces were unable to reduce bacterial adhesion, the presence of fluoro-groups can still provide a means for increasing the hydrophobicity of the corresponding resin composite, thereby altering the hydrophobic nature of the adsorbed protein layer and potentially modulating hydrolytic degradation in the wet oral environment. The latter findings warrant ongoing studies with these materials including studying the nature of bacterial derived esterase activities as a result of the influence that the fluorinated materials had on the protein interaction with monomer components and their polymers.

## 5. Conclusions

We have shown that incorporation of fluoro-chemistry into the monomers is not sufficient to eliminate protein and bacterial adhesion onto polymerized resins. However, such chemistry can be used to alter the nature of the interactions of proteins with these polymers, and specifically influence the nature of hydrophobic groups presented at the surface from the materials and the adhered proteins. This combination of fluorinated and urethane chemistry influences the nature of monomer stability against hydrolytically active protein catalysts, which may ultimately improve the longevity of polymerized composites generated from these monomers, when applied within the oral environment.

## Figures and Tables

**Table 1:** Contact angle (°) measurements are reported with ±SD for films before and after exposure to albumin (see nomenclature for chemical structures in Figure 1). * denotes significance difference (*p* < 0.05) from pre-incubation value.

Sample	Pre-Incubation			Post-Incubation with BSA		
	Advancing	Receding	Hysteresis	Advancing	Receding	Hysteresis
pF_3_LHB	85 (±3)	49 (±4)	36	86 (±4)	46 (±7)	40
pF_4_LHB	88 (±3)	49 (±4)	39	83 (±2) *	45 (±3) *	38
pF_6_LHB	86 (±2)	50 (±4)	36	85 (±3)	45 (±3) *	40
pF_3_LHiB	85 (±3)	49 (±5)	36	81 (±2) *	48 (±4)	33
pF_3_LHH	84 (±2)	49 (±4)	35	81 (±2) *	48 (±2)	33
pF_4_LHH	84 (±3)	51 (±5)	33	83 (±2)	51 (±4)	32
pF_6_LHH	81 (±1)	47 (±4)	34	82 (±2)	43 (±4) *	39
pF_3_LHsH	85 (±3)	49 (±4)	36	81 (±2) *	44 ±5) *	37
pF_3_LHT	86 (±3)	50 (±4)	36	84 (±4)	53 (±4) *	31
pF_4_LHT	81 (±2)	40 (±5)	41	79 (±2) *	39 (±3)	40
pF_6_LHT	83 (±4)	40 (±5)	43	80 (±4)	42 (±4)	38
pBisGMA	72 (±4)	52 (±4)	20	58 (±3) *	36 (±3) *	22
pUDMA	75 (±2)	49 (±4)	26	65 (±4) *	35 (±5) *	30

## Supplementary Materials

The following are available online at www.mdpi.com/2073-4360/9/8/365/s1.
