# Supplementary material for: Biodegradation Studies of Novel Fluorinated Di-Vinyl Urethane Monomers and Interaction of Biological Elements with Their Polymerized Films
Source: Polymers (Basel). 2017 Aug 17;9(8):365. doi: 10.3390/polym9080365 (PMC6418586; doi:10.3390/polym9080365)
Supplement: Supplementary file 1 [file polymers-09-00365-s001.pdf]

## Supplementary Materials

# Biodegradation Studies of Novel Fluorinated Di-Vinyl Urethane Monomers and Interaction of Biological Elements with Their Polymerized Films

Yasaman Delaviz, Meilin Yang and J. Paul Santerre

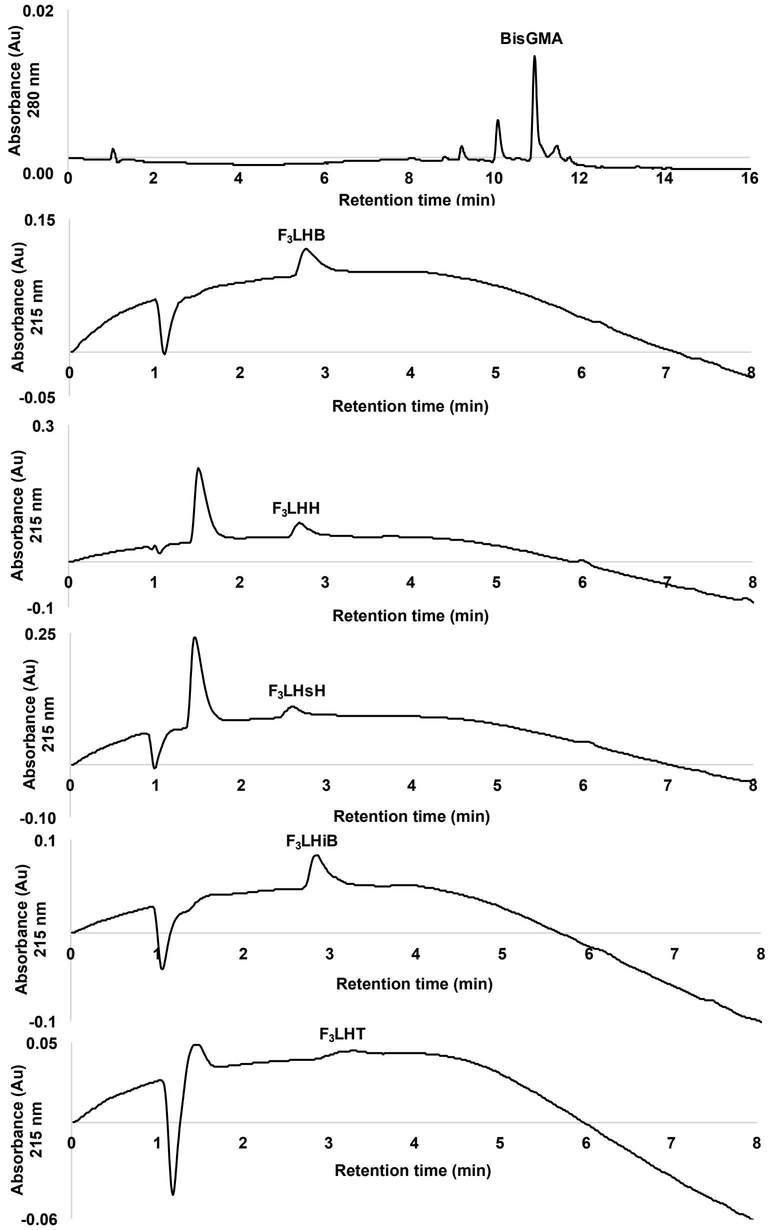


**Figure S1.** HPLC chromatograph for BisGMA and $F_3$LHD monomers at day 0.

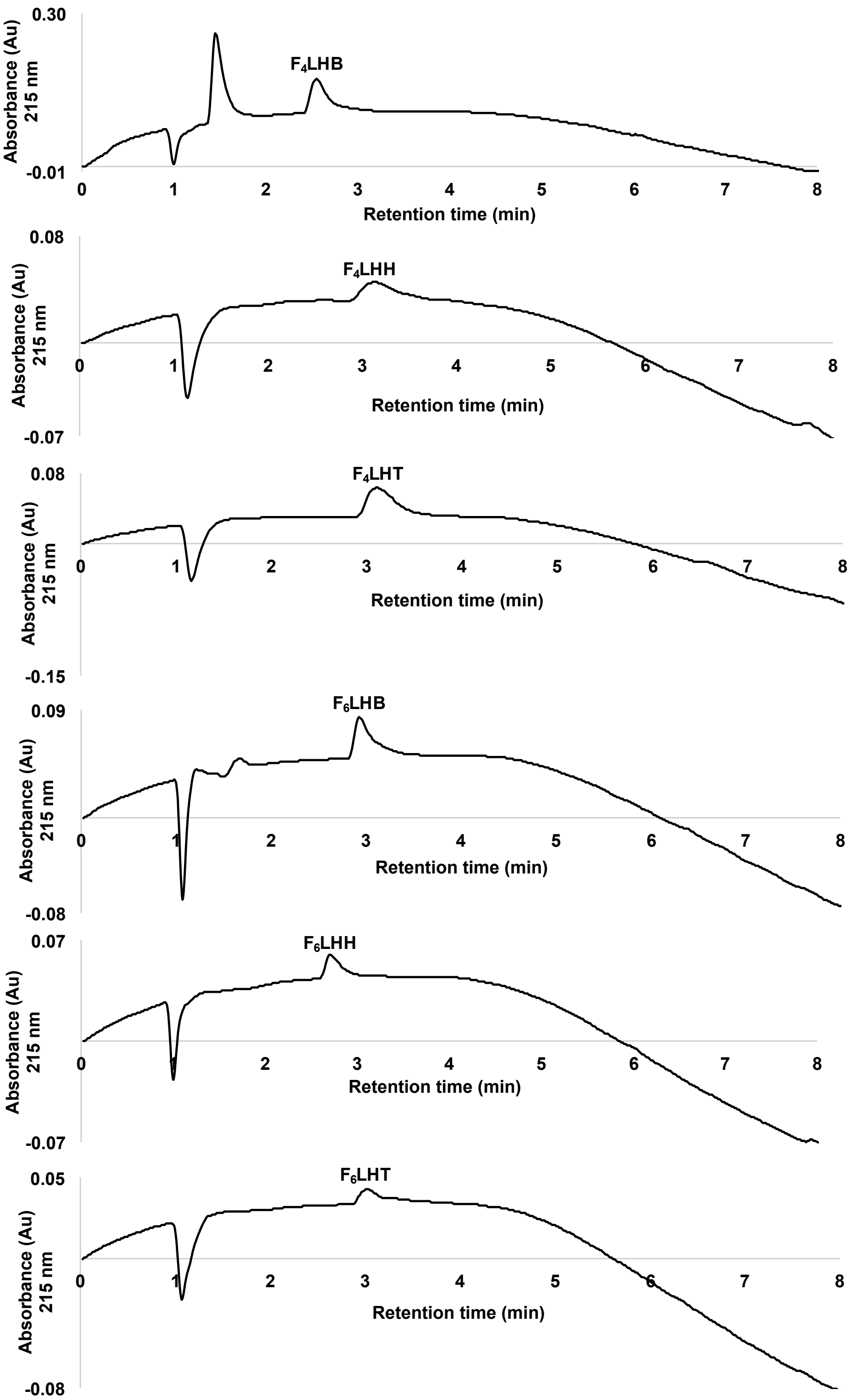


**Figure S2.** HPLC chromatograph for $F_4$LHD and $F_6$LHD monomers at day 0.
